# The causal relationship between steroid hormones and risk of stroke: evidence from a two-sample Mendelian randomization study

**DOI:** 10.1186/s13041-025-01173-2

**Published:** 2025-01-23

**Authors:** Yang Zhang, Miaowen Jiang, Di Wu, Ming Li, Xunming Ji

**Affiliations:** 1 Department of Neurology, Xuanwu Hospital, Capital Medical University, Beijing, China; 2 Xuanwu Hospital, China-America Institute of Neurology, Capital Medical University, No. 45, Changchun Street, Xicheng District, Beijing, China; 3 Beijing Institute of Brain Disorders, Capital Medical University, Beijing, China; 4 Department of Neurosurgery, Xuanwu Hospital, Capital Medical University, No. 45, Changchun Street, Xicheng District, Beijing, China

**Keywords:** Steroid hormones, Stroke, Ischemic stroke, Small vessel stroke, Mendelian randomization study, Single nucleotide polymorphisms

## Abstract

It is unclear how steroid hormones contribute to stroke, and conducting randomized controlled trials to obtain related evidence is challenging. Therefore, Mendelian randomization (MR) technique was employed in this study to examine this association. Through genome-wide association meta-analysis, the genetic variants of steroid hormones, including testosterone/17β-estradiol (T/E2) ratio, aldosterone, androstenedione, progesterone, and hydroxyprogesterone, were acquired as instrumental variables. Analysis was done on the impact of these steroid hormones on the risk of stroke subtypes. The T/E2 ratio was associated to an elevated risk of small vessel stroke (SVS) according to the inverse variance weighted approach which was the main MR analytic technique (OR, 1.23, 95% CI: 1.05–1.44, *p* = 0.009). These findings were solid since no heterogeneity nor horizontal pleiotropy were found. The causal association between T/E2 and SVS was also confirmed in the replication study (*p* = 0.009). Nevertheless, there was no proof that other steroid hormones increased the risk of stroke. According to this study, T/E2 ratio and SVS are causally related. However, strong evidence for the impact of other steroid hormones on stroke subtypes is still lacking. These findings may be beneficial for developing stroke prevention strategies from steroid hormones levels.

**Supplementary Information:**

The online version contains supplementary material available at 10.1186/s13041-025-01173-2.

## Introduction

Steroid hormones are tetracyclic aliphatic hydrocarbon compounds with a cyclopentanopolyhydrophenanthrene ring, including testosterone (T), 17β-estradiol (E2), aldosterone (Aldo), androstenedione (A4), progesterone (P4), and hydroxyprogesterone (17-OHP) [1]. These hormones are mainly biosynthesized from cholesterol in the adrenal cortex and gonads [2]. They play roles in various physiological functions, such as regulating blood pressure, controlling sexual function, and immunoregulation [3–5]. There is a complex synthetic network among steroid hormones. When the concentration of one component becomes abnormal due to diseases of the adrenal cortex and gonads, the dynamic balance of steroid hormones in the body may be disrupted, leading to significant changes in their levels. Moreover, steroid hormone secretion disorders can affect other crucial systems, such as the cardiovascular system [6, 7]. For example, primary aldosteronism will lead to water and sodium retention, which will lead to secondary hypertension, and increase the incidence of cardiovascular diseases [6].

Stroke, including ischemic and hemorrhagic types, is a common cardiovascular disease and a major cause of death and disability worldwide, with a continuous upward trend [8, 9]. Recently, hypertension, smoking, alcohol consumption, diabetes, and hyperlipidemia have been identified as stroke risk factors. However, due to the various pathologies of stroke, identifying risk factors is complex [10]. Many observational studies have found that steroid hormone disorders significantly influence the cardiovascular system and contribute to stroke [11, 12]. However, confounders and reverse causality are two main biases that might affect observational research. Randomized controlled trials investigating this association are also difficult to perform due to ethical concerns. Furthermore, most epidemiological data have not examined the connections between stroke subtypes and steroid hormones. Therefore, determining the specific causal impact of different hormones on stroke subtypes can lead to more precise prevention strategies.

In the Mendelian randomization (MR) technique, genetic variants are used as instrumental variables (IVs) to refer to exposure factors, which applies the rule of independent assortment to assess the causal influence of exposure on the outcome. MR generally avoids the effects of confounders and reverse causality [13]. Additionally, single nucleotide polymorphisms (SNPs) can be employed to infer causality in MR research based on data from genome-wide association meta-analysis (GWAMA). Thus, using summary GWAMA data, we performed two-sample MR analyses in this work to investigate the genetic association of steroid hormones and stroke subtypes.

## Methods

### Study design

**Fig. 1:**
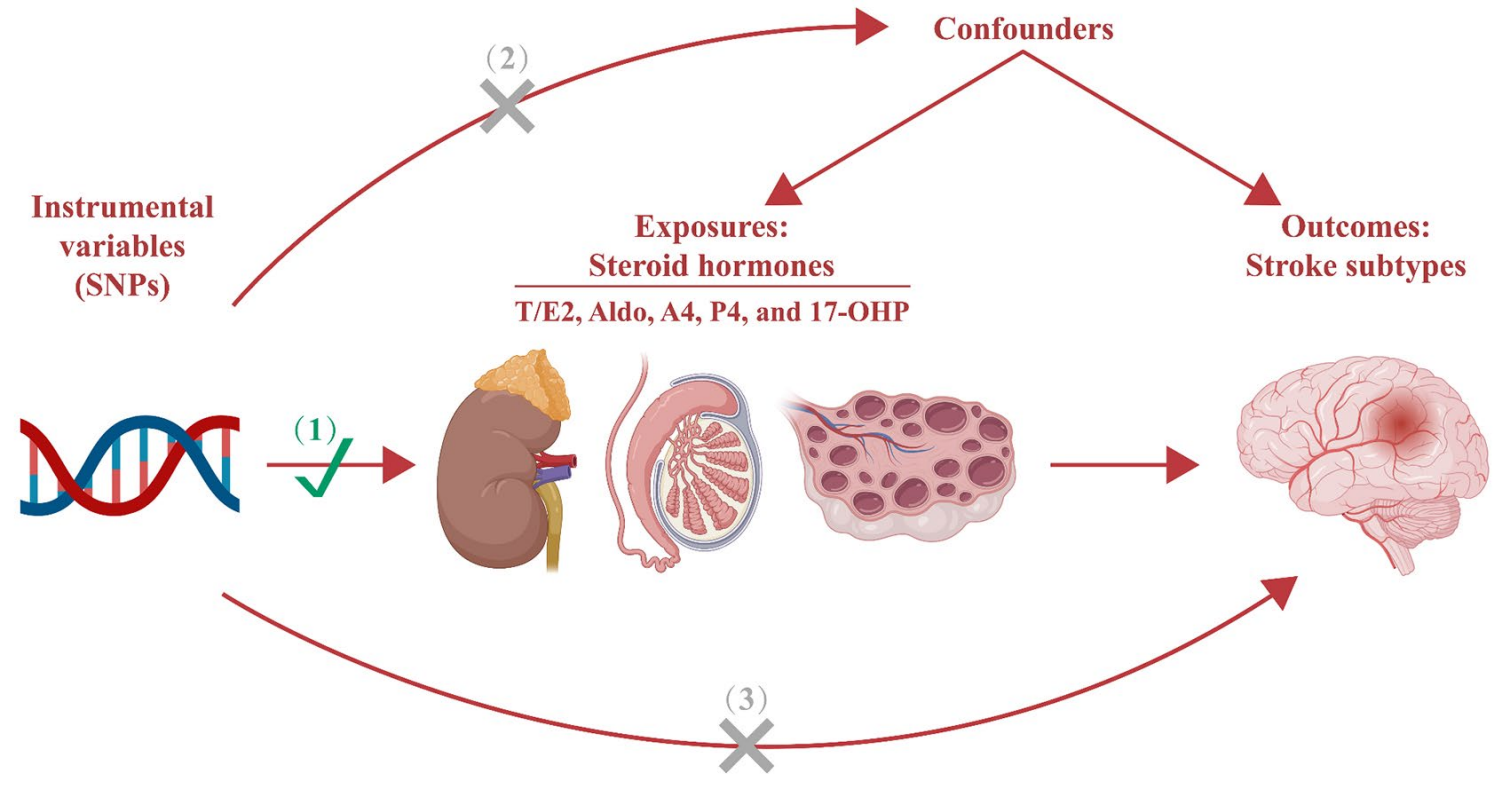
Mendelian randomization model of the present study. The design is based on the assumptions that the instrumental variables (1) are associated with steroid hormones, (2) are not associated with confounders, and (3) influence stroke subtypes only through steroid hormones. SNPs, single nucleotide polymorphisms

**Fig. 2:**
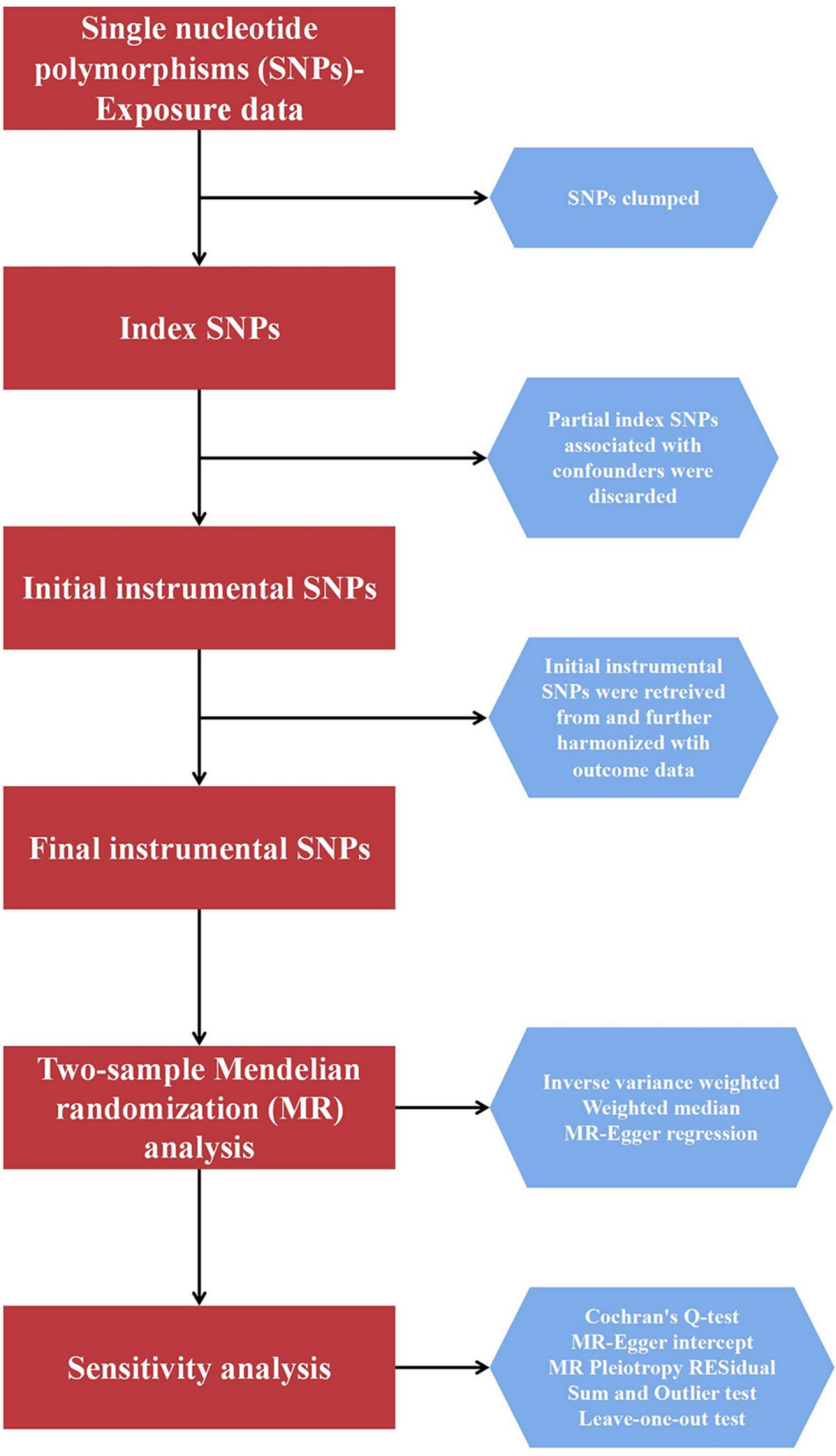
Workflow of the present Mendelian randomization study

In this MR study, genetic variants associated with steroid hormones were used as IVs to examine the causal role of steroid hormones in stroke subtypes. IVs should satisfy the following assumptions: (1) exposure is strongly related to IVs; (2) confounders are not related to IVs; and (3) exposure alone is how IVs influence the result (Fig. 1) [14]. The study process of this MR analysis is presented in Fig. 2. In the original trials, appropriate ethical approval and informed consent from the patients were obtained, and no additional ethical approval was required.

### Data source

Table S1 displays the participant characteristics from the datasets on steroid hormones and stroke. The participants were matched by ethnicity but not by age or gender. The steroid hormone data were obtained from a recent GWAMA conducted in individuals of European descent [15]. This study produced GWAMA data with adjustments for sex, age, and log-transformed BMI from two separate investigations, LIFE-Adult (1481 cases) [16] and LIFE-Heart (2068 cases) [17], concerning one steroid hormone ratio (T/E2) and the levels of four steroid hormones, including Aldo, A4, P4, and 17-OHP.

Data on 8 stroke subtypes from different GWAMA studies were used as outcome variables. The MEGASTROKE consortium produced the genetic information for any ischemic stroke (AIS, 4,217 cases and 406,111 controls) and its subtypes (large artery stroke (LAS; *n* = 7,193), cardioembolic stroke (CES; *n* = 5,386), and small vessel stroke (SVS; *n* = 4,373)) [18]. Patients with LAS exhibit clinical and brain imaging manifestations of either obvious stenosis or occlusion of the large cerebral artery or cortical branch, possibly caused by atherosclerosis. CES includes patients who may experience arterial occlusion due to an embolus arising in the heart. SVS usually refers to patients with lacunar infarction.

There are two types of hemorrhagic stroke: intracerebral hemorrhage (ICH) and subarachnoid hemorrhage (SAH). The International Stroke Genetics Consortium released European GWAMA consisting 1,545 ICH cases (664 lobar and 881 deep) and 1,481 controls, as the source of the genetic information for ICH [19]. A recent European GWAMA of cerebral aneurysms, which included 5,140 SAH patients caused by cerebral aneurysm rupture and 71,934 controls, provided data on SAH since non-traumatic SAH is predominantly caused by aneurysm rupture [20].

To further verify the reliability of the results, summary statistics for SVS in individuals of European ancestry (6,030 cases, 219,389 controls) were extracted from another GWAMA conducted by Traylor et al. in 2021 for replication analysis [21].

### Selection of instrumental variables

Similar to other MR research, the threshold was set at *p* < 1 × 10^− 5^ in order to obtain sufficient IVs [22]. This was necessary because there were insufficient SNPs associated with exposure when the threshold was set as 5 × 10^− 8^. All SNPs with locus-wide significance were then clumped based on linkage disequilibrium threshold (r^2^ = 0.001) and a distance threshold (10,000 kb) to create index SNPs [23]. Some index SNPs associated with potential risk factors were removed (Table S2) following a search of all index SNPs in PhenoScanner V2, which provides detailed information on the relationship between genotype and phenotype [24]. The initial instrumental SNPs were thus obtained (15 SNPs for T/E2, 16 SNPs for Aldo, 15 SNPs for A4, 28 SNPs for P4, and 10 SNPs for 17-OHP). Next, F-statistics were used to indicate the IVs’ strength [F = (N-K-1) × R^2^ / K / (1-R^2^); R^2^: the percentage of variation accounted for the relationship between the IVs; K: the number of IVs; N: the number of samples]. All remaining SNPs had F-statistics larger than 10 [25]. Finally, SNPs were picked up from and harmonized with outcome data to generate the final instrumental SNPs which were not associated with stroke subtypes and did not contain any mismatched or palindromic SNPs. The characteristics of the final instrumental SNPs related to steroid hormones and stroke subtypes are displayed in Table S3.

### Statistical analyses

Three distinct methods were used in the present MR study: inverse-variance weighted (IVW), weighted median, and MR-Egger regression. Among all MR techniques, IVW is the most statistically powerful standard MR algorithm. It provides MR estimate by combining each Wald ratio of instrumental SNPs [26]. If invalid IVs account for less than 50% of the weight, the weighted median can yield accurate estimations [27]. MR-Egger regression is mainly used to detect and explain directional pleiotropy bias [28]. We also displayed the genetic associations of steroid hormones with stroke subtypes using scatterplots of genetic variants.

Furthermore, sensitivity analyses, which included tests for heterogeneity (IVs can affect multiple aspects of exposure factors) and horizontal pleiotropy (IVs can directly affect outcomes without exposure factors), were conducted to identify various violations of assumptions. Cochran’s Q test was used in the IVW approach to quantify heterogeneity. There was heterogeneity in the causal effects across all SNPs when the *p* value of the Cochran’s Q statistic was less than 0.05 [29]. Horizontal pleiotropy was tested by the intercept test of MR-Egger and the MR-pleiotropy residual sum and outlier (MR-PRESSO) test. First, horizontal pleiotropy was shown by a non-zero MR-Egger regression intercept [28]. Second, the global and outlier test in MR-PRESSO could identify horizontal pleiotropic outliers and adjust pleiotropy by removing outliers [30]. Additionally, leave-one-out analysis was employed to see whether a particular SNP had an impact on the overall MR estimate.

The statistically significant *p* value criterion was chosen at 0.01 following the Bonferroni correction because this MR study covered five exposures. To determine the suggestive causal association, a *p* value between the Bonferroni-corrected significance level and the conventional significance threshold (0.05) was employed.

R software (version 4.2.1) and its companion R package, TwoSampleMR (version 0.5.6), were used to perform all of the statistical studies indicated above.

## Results

### Causal effect of T/E2 ratio on stroke subtypes

**Fig. 3:**
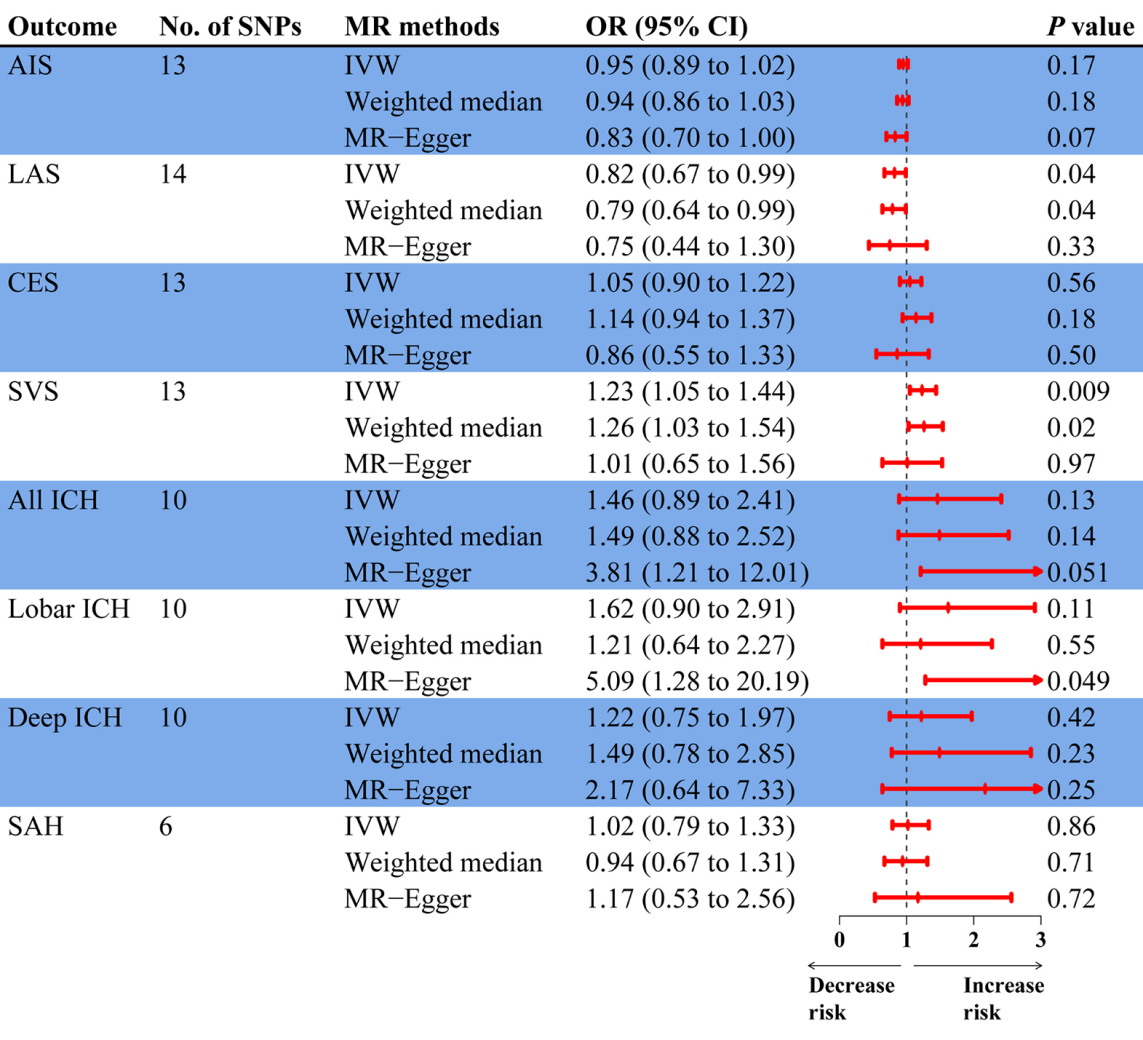
MR association of genetically determined T/E2 ratio with stroke subtypes. MR, Mendelian randomization; T/E2, testosterone/17β-estradiol; AIS, any ischemic stroke; LAS, large artery stroke; CES, cardioembolic stroke; SVS, small vessel stroke; ICH, intracerebral hemorrhage; SAH, subarachnoid hemorrhage; SNPs, single nucleotide polymorphisms; IVW, inverse variance weighted; OR, odd ratio; CI, confidence interval

**Fig. 4:**
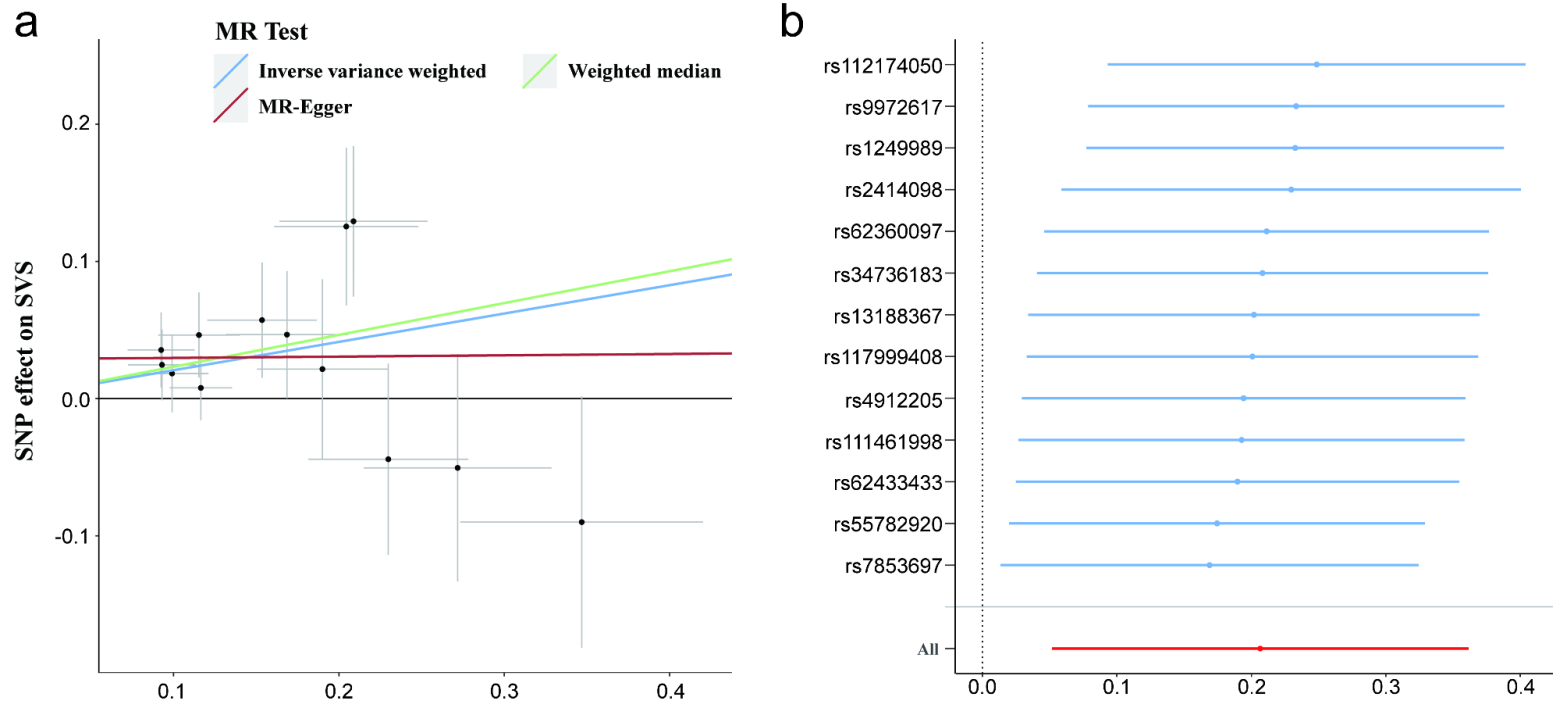
Scatter plot (**a**) and leave-one-out test (**b**) for genetically determined T/E2 ratio and risk of SVS. T/E2, testosterone/17β-estradiol; SVS, small vessel stroke; MR, mendelian randomization; SNP, single nucleotide polymorphism

According to the IVW technique and the weighted median method, the T/E2 ratio had an obvious causal influence on SVS (OR, 1.23, 95% CI: 1.05–1.44, *p* = 0.009) and a suggestive causal effect on LAS (OR: 0.82, 95% CI: 0.67–0.99, *p* = 0.04) (Fig. 3). The estimate was directionally compatible with IVW and weighted median analysis, despite the fact that the MR-Egger study did not yield significant results (Figs. 3 and 4a, and Figure S1). No causal role of the T/E2 ratio was detected in other stroke subtypes. Furthermore, horizontal pleiotropy and heterogeneity were not detected by sensitivity analysis, confirming the MR analysis’s robustness (Table S4). Finally, the IVW point estimate was not dominated by any particular SNP, as the leave-one-out analysis verified. (Fig. 4b and Figure S1). Moreover, there was no gender differences in this association (Table S5).

### Causal effect of Aldo on stroke subtypes

The IVW results showed that Aldo had no causal influence on any stroke subtype, as seen in Figure S2 (all *p* > 0.05). The evidence from the weighted median and MR-Egger techniques was comparable with the IVW findings for the association of Aldo with stroke subtypes, with the exception of the weighted median analysis’s suggestive significance for the causal influence of Aldo on LAS (*p* = 0.02) (Figure S2). Sensitivity analysis showed no heterogeneity, except for the impact of Aldo on AIS (Q, 30.85; *p* = 0.009). Neither the MR-Egger intercept nor the MR-PRESSO global test indicated directional pleiotropy for Aldo, apart from the relationship between Aldo and AIS (MR-PRESSO global test, *p* = 0.01) (Figure S2). The MR-PRESSO outlier test identified rs74911566 as an outlier SNP. After correcting for the outlier, the results remained non-significant (Table S6). Finally, the leave-one-out study demonstrated that the risk assessment of genetically predicted Aldo and the risk of stroke subtypes were largely consistent (Figure S3).

### Causal effect of A4 on stroke subtypes

A4 showed no significant causal relationship with any stroke subtypes as detected by three MR method (all *p* > 0.05) (Figure S4). According to Cochran’s Q test, there was no heterogeneity in these results except for SAH (Q, 11.17; *p* = 0.02) and no pleiotropy was detected. More details are provided in Figure S4. The dependability of the data between A4 and all stroke subtypes was further validated by the leave-one-out analysis. (Figure S5).

### Causal effect of P4 on stroke subtypes

A possible correlation between circulating P4 levels and the risk of deep ICH was found by the IVW study (OR: 0.59, 95% CI: 0.35–0.99, *p* = 0.048). However, there was no proof that P4 and other stroke subtypes were causally related (*p* > 0.05) (Figure S6). There was no discernible correlation between P4 and other stroke subtypes detected by weighted median or MR-Egger approaches (all *p* > 0.05) (Figure S6). Some heterogeneity was found between P4 and SVS (Q, 41.02; *p* = 0.02) as well as P4 and lobar SAH (Q, 27.19; *p* = 0.01). Though there was no directional pleiotropy between P4 and any of the stroke subtypes according to the MR-Egger intercept test, the MR-PRESSO global test produced a significant result (*p* = 0.02). One SNP, rs117848367, was identified as an outlier by the MR-PRESSO outlier test in the relationship between P4 and SVS. However, further outlier-corrected results were similar to those before correction (Table S7). Moreover, the causal association between P4 and any stroke subtypes was not demonstrated by a single IV, according to the leave-one-out analysis (Figure S7).

### Causal effect of 17-OHP on stroke subtypes

There were no significant results from any MR methods to support the causal impact of 17-OHP on stroke subtypes (all *p* > 0.05) (Figure S8). According to sensitivity analysis, the results were robust and not affected by heterogeneity or directional pleiotropy (Figure S8). Furthermore, the robustness of the data between 17-OHP and each stroke subtype was further validated using the leave-one-out approach (Figure S9).

### Replication analysis

Since SVS was found to have a causal association with the T/E2 ratio, summary statistics for SVS from another GWAMA were used for replication analysis (Figure S10). Although the other two MR analyses did not provide significant causal associations between the T/E2 ratio and SVS in the replication stage, the estimate conducted from the IVW approach agreed with primary findings (OR, 1.23, 95% CI: 1.02–1.47, *p* = 0.009). No causal relationship was found between the other four hormone exposure factors and SVS. Additionally, sensitivity analysis showed no heterogeneity, directional pleiotropy, or outliers. The causal relationship between each steroid hormone and SVS was not driven by any specific IV, according to the leave-one-out analysis (Figure S11).

## Disccusion

After comprehensively evaluating the causal association of different steroid hormones abd various stroke subtypes using data from GWAMA, the current study indicated that an increasing T/E2 ratio had a positive causal correlation with SVS risk but not with other stroke subtypes. This finding was confirmed in the replication stage. Moreover, there was no gender difference in this relationship. The present study suggests that an increase in serum T concentration or a decrease in serum E2 concentration will raise the risk of SVS. However, the study did not support a role for any other steroid hormones in stroke risk.

There is currently no solid evidence about how T affects the risk of stroke. The largest related study included over 80,000 male veterans who had filled a T prescription and over 120,000 who had not [31]. The composite outcome of IS, acute myocardial infarction, or venous thromboembolic disease did not correlate with intramuscular T therapy. However, other research has revealed that fluctuating T levels during a man’s life, with higher levels in young men and lower levels in elderly men, may increase the risk of IS [32, 33]. The effect of E2 levels on stroke is also inconclusive. Alonso de Leciñana et al. studied postmenopausal women in a multicenter, age-matched, case-control study, which indicated that longer-term ovarian E2 exposure might prevent noncardioembolic IS [34]. However, an analysis of data from a postmenopausal women’s nested case-control study from the Nurses’ Health Study by Hu et al. concluded that total or free E2 levels had no role in mediating the risk of IS [35]. Moreover, Abbott et al.‘s follow-up analysis of older men who took part in the Honolulu-Asia Aging analysis revealed that elevated blood E2 levels may be related to an elevated risk of stroke in older men [36].

The conflicting outcomes from these observational studies may be resulted from small sample sizes and the presence of confounding factors that are difficult to eliminate. Additionally, the small sample sizes prevent further analysis of stroke subtypes. Furthermore, it is not obvious if the association between the risk of stroke and circulating levels of T and E2 shown in observational studies is causative or an example of reverse causality. Furthermore, it is not obvious if the association between the risk of stroke and circulating levels of T and E2 shown in observational studies is causal or an example of reverse causality [37–39]. To get over the drawbacks of observational research and examine the relationship between the T/E2 ratio and stroke subtypes, we therefore carried out an MR investigation. According to our research, the T/E2 ratio may increase the risk of SVS.

Several potential mechanisms could support our findings. There is robust preclinical evidence of the neuroprotective and anti-inflammatory properties of E2. For example, Ghisletti et al. indicated that E2 could bind to E2 receptor α isoform to inhibit NF-κB signaling pathway and attenuate neuroinflammation. This mechanism involves PI3K activation [40, 41]. However, the role of T in stroke is not clear. Many conflicting findings have been reported in studies evaluating the effects of T on atherosclerosis and lipid metabolism. T has been indicated to increase blood viscosity and platelet activation, promoting thrombosis. Additionally, T could improve renal salt and water retention [42, 43]. Interestingly, by converting to E2 via aromatase in endothelial cells, T may reduce the expression of vascular cell adhesion molecule-1 [44]. This evidence suggests that E2 is more likely to inhibit the occurrence of stroke, whereas the function of T remains unclear. Furthermore, one study has indicated that T could impair microvascular endothelial function [45], and another study showed that compared with large vessels, T was more likely to damage small vessels [46]. This information could explain why an elevated T concentration seems to be a unique risk factor for SVS. Although our research has theoretical support, more studies are needed to reinforce this perspective.

The following limitations should be mentioned in the MR analysis’s findings. First, our data do not support a significant influence of steroid hormones other than the T/E2 ratio on stroke subtype risk. The reason for this phenomenon maybe the sample size of exposure was too small so there was insufficient SNPs to obtain significant MR results. Thus, the sample size of GWAMA about steroid hormones should be increased in subsequent research to collect more reliable IVs for additional validation and more conclusive results. Second, the association between Aldo, A4, and P4 and the risk of stroke subtypes should be assessed with care because potential heterogeneity, pleiotropy, or outliers could be present. Lastly, there was a restriction to extrapolating the discovered causal relationships to other groups with different genetic backgrounds because only data from summary statistics for people with European ancestry were included.

In conclusion, a causal association between the T/E2 ratio and SVS was found by MR analysis. However, strong evidence for the impact of other steroid hormones on stroke subtypes is still lacking. According to our findings, an increase in T or a decrease in E2 during continuous monitoring of steroid hormone levels in the blood may be a biomarker for the occurrence of SVS. Therefore, patients with diseases that may experience this trend, such as men with testicular tumors and menopausal women, should be particularly aware of the possibility of developing SVS and should be closely followed up. Further investigation is warranted to explore the specific mechanisms of T and E2 in the pathogenesis of SVS.

## Electronic supplementary material

Below is the link to the electronic supplementary material.

Supplementary Material 1

Supplementary Material 2

## Data Availability

All data used in our study are publicly available, which could be available from https://www.mdpi.com/article/10.3390/metabo11110738/s1 (steroid hormones); http://www.megastroke.org/download.html (ischemic stroke); https://cd.hugeamp.org/ downloads.html (intracerebral hemorrhage); 10.6084/m9.fifigshare.11303372 (subarachnoid hemorrhage).

## Abbreviations

E2: 17β-estradiol
Aldo: Aldosterone
A4: Androstenedione
AIS: Any ischemic stroke
CES: Cardioembolic stroke
GWAMA: Genome-wide association meta-analysis
17-OHP: Hydroxyprogesterone
IVs: Instrumental variables
ICH: Intracerebral hemorrhage
IVW: Inverse-variance weighted
LAS: Large artery stroke
MR: Mendelian randomization
MR-PRESSO: MR-pleiotropy residual sum and outlier
P4: Progesterone
SNPs: Single nucleotide polymorphisms
SVS: Small vessel stroke
SAH: Subarachnoid hemorrhage
T: Testosterone
