# Supplementary material for: The causal relationship between steroid hormones and risk of stroke: evidence from a two-sample Mendelian randomization study
Source: Mol Brain. 2025 Jan 23;18:6. doi: 10.1186/s13041-025-01173-2 (PMC11758733; doi:10.1186/s13041-025-01173-2)
Supplement: Supplementary file 1 — Supplementary Material 1 [file 13041_2025_1173_MOESM1_ESM.pdf]

Supplementary Files

Figure S1. Scatter plot and leave-one-out test for genetically determined T/E2 ratio and risk of stroke subtypes.

Figure S2. MR estimates from each method of assessing the causal effects of Aldo on the risk of stroke subtypes.

Figure S3. Scatter plot and leave-one-out test for genetically determined Aldo and risk of stroke subtypes.

Figure S4. MR estimates from each method of assessing the causal effects of A4 on the risk of stroke subtypes.

Figure S5. Scatter plot and leave-one-out test for genetically determined A4 and risk of stroke subtypes.

Figure S6. MR estimates from each method of assessing the causal effects of P4 on the risk of stroke subtypes.

Figure S7. Scatter plot and leave-one-out test for genetically determined P4 and risk of stroke subtypes.

Figure S8. MR estimates from each method of assessing the causal effects of 17-OHP on the risk of stroke subtypes.

Figure S9. Scatter plot and leave-one-out test for genetically determined 17-OHP and risk of stroke subtypes.

Figure S10. MR estimates from each method of assessing the causal effects of steriod on the risk of SVS in replicated stage.

Figure S11. Scatter plot and leave-one-out test for genetically determined steroid harmonies and risk of SVS in replicated stage.

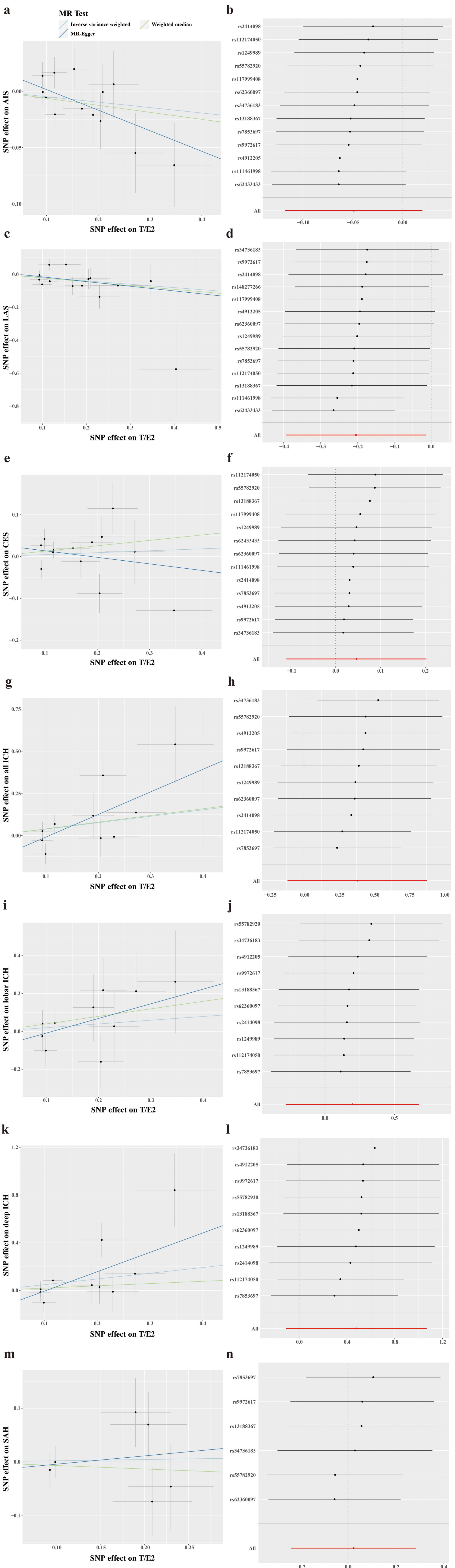

Figure S1. Scatter plot and leave-one-out test for genetically determined T/E2 ratio and risk of stroke subtypes. (a, b) AIS, any ischemic stroke; (c, d) LAS, large artery stroke; (e, f) CES, cardioembolic stroke; (g, h) all ICH, intracerebral hemorrhage; (i, j) lobar ICH; (k, l) deep ICH; (m, n) SAH, subarachnoid hemorrhage. T/E2 ratio, testosterone/17β-estradiol ratio; SNP, single nucleotide polymorphism.

Outcome	No. of SNPs	MR methods	OR (95% CI)	MR *P* value	Heterogeneity test (*Q*−statistic, *P*)	MR−Egger (intercept, *P*)	*P* for MR−PRESSO global test
AIS	16	IVW	1.01 (0.91 to 1.12)	0.82	30.85, 0.009	0.02, 0.23	0.01
		Weighted median	0.94 (0.84 to 1.04)	0.23			
		MR−Egger	0.86 (0.65 to 1.13)	0.30			
LAS	16	IVW	0.84 (0.70 to 1.01)	0.06	13.90, 0.53	−0.02, 0.44	0.54
		Weighted median	0.75 (0.58 to 0.96)	0.02			
		MR−Egger	1.01 (0.61 to 1.66)	0.97			
CES	16	IVW	0.95 (0.82 to 1.09)	0.46	14.02, 0.52	0.04, 0.09	0.52
		Weighted median	0.97 (0.79 to 1.19)	0.76			
		MR−Egger	0.68 (0.46 to 1.00)	0.07			
SVS	16	IVW	1.05 (0.85 to 1.30)	0.65	23.29, 0.08	0.02, 0.60	0.08
		Weighted median	0.96 (0.75 to 1.22)	0.72			
		MR−Egger	0.91 (0.51 to 1.63)	0.75			
All ICH	8	IVW	1.08 (0.63 to 1.83)	0.78	5.29, 0.62	0.07, 0.38	0.63
		Weighted median	1.19 (0.60 to 2.38)	0.62			
		MR−Egger	0.52 (0.11 to 2.53)	0.45			
Lobar ICH	8	IVW	0.97 (0.52 to 1.83)	0.93	5.84, 0.56	0.12, 0.23	0.83
		Weighted median	0.81 (0.34 to 1.92)	0.64			
		MR−Egger	0.29 (0.04 to 1.92)	0.24			
Deep ICH	8	IVW	1.30 (0.65 to 2.60)	0.45	3.54, 0.83	0.01, 0.91	0.57
		Weighted median	1.06 (0.43 to 2.60)	0.90			
		MR−Egger	1.16 (0.15 to 8.90)	0.89			
SAH	11	IVW	0.92 (0.74 to 1.14)	0.43	5.69, 0.84	−0.005, 0.87	0.85
		Weighted median	0.91 (0.68 to 1.22)	0.54			
		MR−Egger	0.96 (0.53 to 1.74)	0.90			

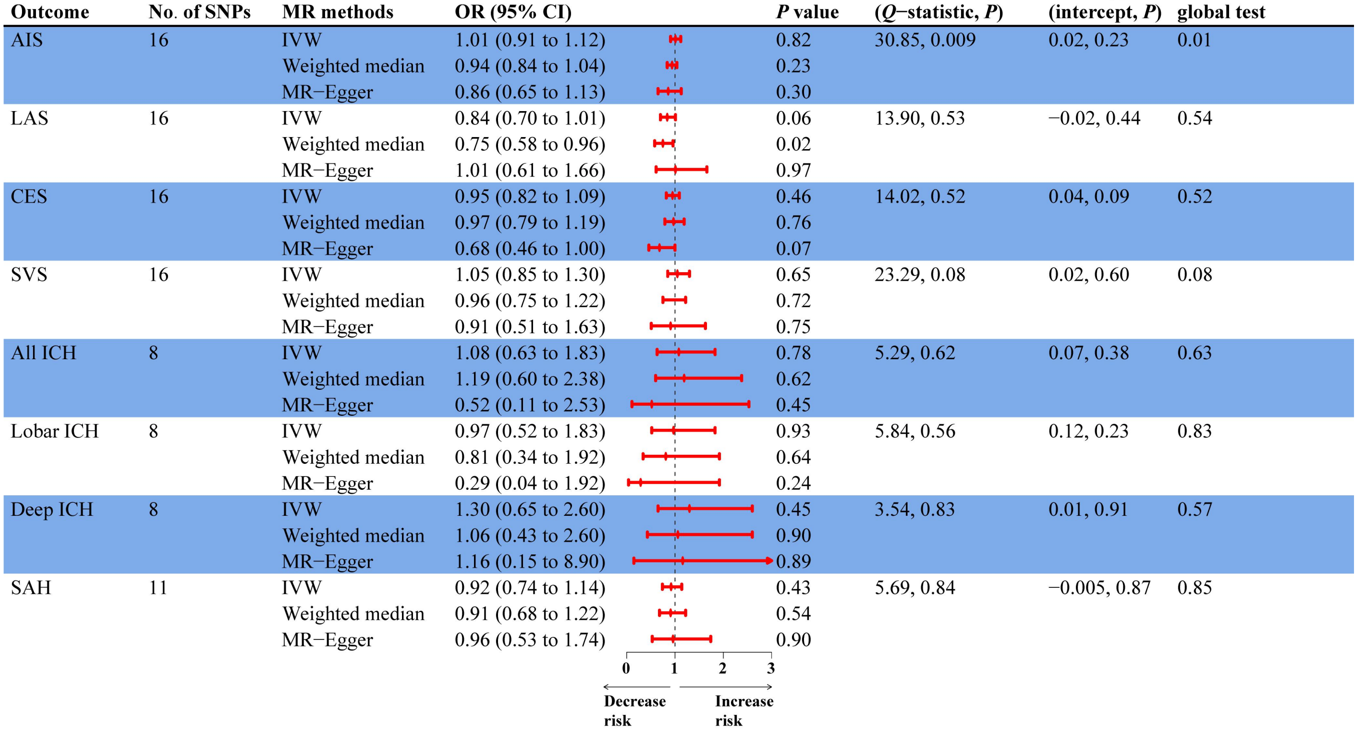

Figure S2. MR estimates from each method of assessing the causal effects of Aldo on the risk of stroke subtypes. Aldo, aldosterone; AIS, any ischemic stroke; LAS, large artery stroke; CES, cardioembolic stroke; SVS, small vessel stroke; ICH, intracerebral hemorrhage; SAH, subarachnoid hemorrhage; SNPs, single nucleotide polymorphisms; MR, Mendelian randomization; IVW, inverse variance weighted; OR, odd ratio; CI, confidence interval; MR-PRESSO, MR pleiotropy residual sum and outlier.

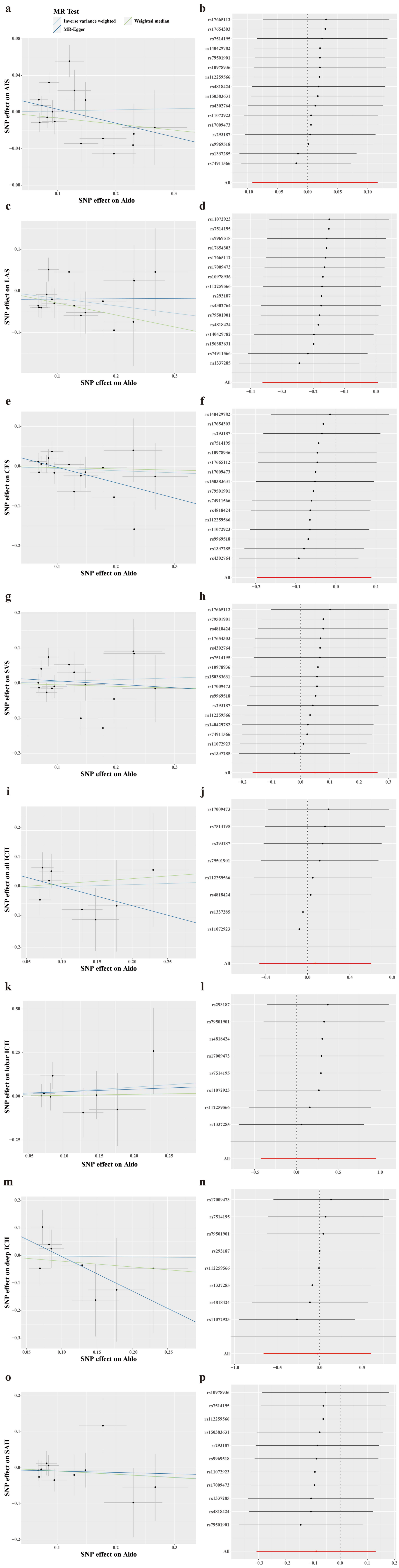

Figure S3. Scatter plot and leave-one-out test for genetically determined Aldo and risk of stroke subtypes. (a, b) AIS, any ischemic stroke; (c, d) LAS, large artery stroke; (e, f) CES, cardioembolic stroke; (g, h) SVS, small vessel stroke; (i, j) all ICH, intracerebral hemorrhage; (k, l) lobar ICH; (m, n) deep ICH; (o, p) SAH, subarachnoid hemorrhage. Aldo, aldosterone; SNP, single nucleotide polymorphism.

Outcome	No. of SNPs	MR methods	OR (95% CI)	*P* value	Heterogeneity test (*Q*−statistic, *P*)	MR−Egger (intercept, *P*)	*P* for MR−PRESSO global test
AIS	14	IVW	0.97 (0.86 to 1.09)	0.61	13.23, 0.43	0.008, 0.45	0.43
		Weighted median	0.98 (0.82 to 1.16)	0.79			
		MR−Egger	0.87 (0.66 to 1.16)	0.38			
LAS	14	IVW	1.15 (0.80 to 1.66)	0.45	19.92, 0.10	0.05, 0.15	0.11
		Weighted median	1.23 (0.80 to 1.88)	0.34			
		MR−Egger	0.64 (0.28 to 1.46)	0.31			
CES	14	IVW	0.97 (0.77 to 1.23)	0.83	8.62, 0.80	0.003, 0.86	0.81
		Weighted median	1.02 (0.74 to 1.41)	0.89			
		MR−Egger	0.93 (0.53 to 1.64)	0.81			
SVS	14	IVW	0.82 (0.62 to 1.07)	0.14	12.92, 0.46	−0.01, 0.70	0.47
		Weighted median	0.82 (0.57 to 1.20)	0.32			
		MR−Egger	0.92 (0.48 to 1.80)	0.82			
All ICH	6	IVW	1.72 (0.77 to 3.86)	0.19	2.35, 0.80	0.07, 0.45	0.81
		Weighted median	1.58 (0.60 to 4.17)	0.36			
		MR−Egger	0.69 (0.07 to 6.91)	0.77			
Lobar ICH	6	IVW	1.32 (0.48 to 3.63)	0.59	5.56, 0.35	0.11, 0.33	0.99
		Weighted median	0.94 (0.26 to 3.32)	0.92			
		MR−Egger	0.30 (0.02 to 4.89)	0.45			
Deep ICH	7	IVW	2.45 (0.88 to 6.80)	0.09	0.93, 0.99	−0.04, 0.71	0.38
		Weighted median	2.10 (0.57 to 7.68)	0.26			
		MR−Egger	4.00 (0.28 to 57.63)	0.36			
SAH	5	IVW	0.87 (0.41 to 1.85)	0.72	11.17, 0.02	−0.02, 0.81	0.05
		Weighted median	1.11 (0.60 to 2.07)	0.74			
		MR−Egger	1.14 (0.12 to 10.55)	0.91			

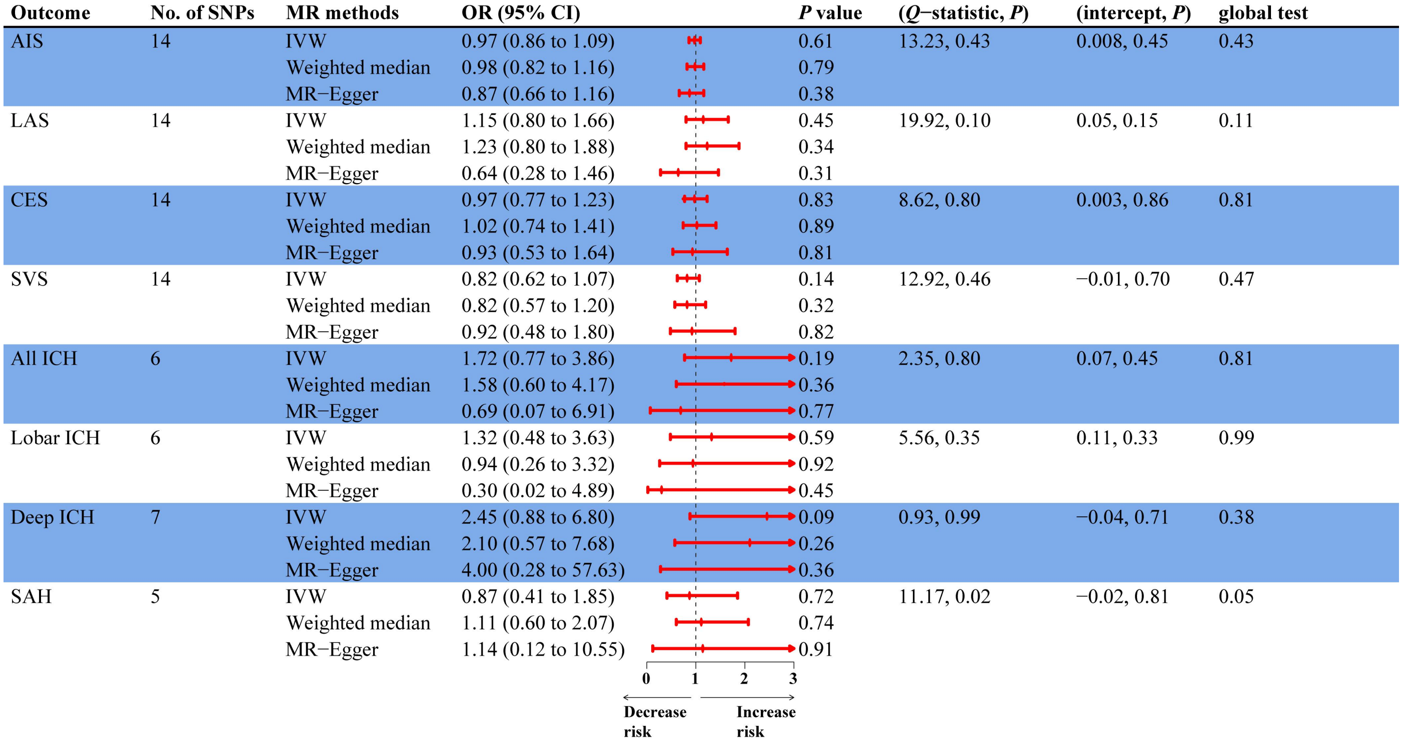

Figure S4. MR estimates from each method of assessing the causal effects of A4 on the risk of stroke subtypes. A4, androstenedione; AIS, any ischemic stroke; LAS, large artery stroke; CES, cardioembolic stroke; SVS, small vessel stroke; ICH, intracerebral hemorrhage; SAH, subarachnoid hemorrhage; SNPs, single nucleotide polymorphisms; MR, Mendelian randomization; IVW, inverse variance weighted; OR, odd ratio; CI, confidence interval; MR-PRESSO, MR pleiotropy residual sum and outlier.

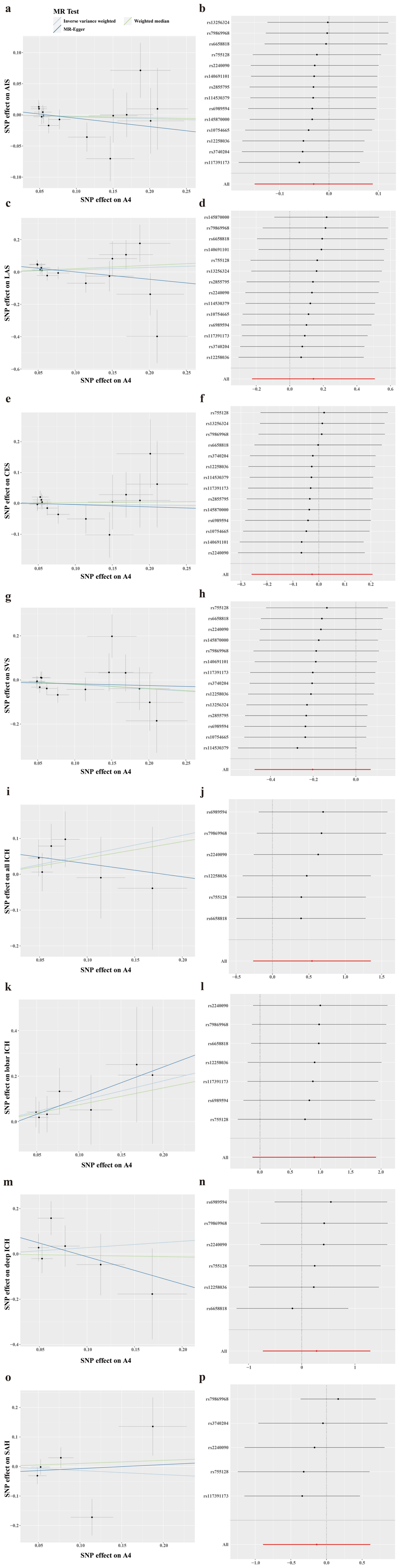

Figure S5. Scatter plot and leave-one-out test for genetically determined A4 and risk of stroke subtypes. (a, b) AIS, any ischemic stroke; (c, d) LAS, large artery stroke; (e, f) CES, cardioembolic stroke; (g, h) SVS, small vessel stroke; (i, j) all ICH, intracerebral hemorrhage; (k, l) lobar ICH; (m, n) deep ICH; (o, p) SAH, subarachnoid hemorrhage. A4, androstenedione; SNP, single nucleotide polymorphism.

Outcome	No. of SNPs	MR methods	OR (95% CI)	*P* value	Heterogeneity test (*Q*−statistic, *P*)	MR−Egger (intercept, *P*)	*P* for MR−PRESSO global test
AIS	27	IVW	1.00 (0.94 to 1.06)	0.94	18.84, 0.84	0.006, 0.48	0.85
		Weighted median	1.02 (0.94 to 1.11)	0.59			
		MR−Egger	0.96 (0.85 to 1.08)	0.52			
LAS	27	IVW	1.06 (0.90 to 1.24)	0.50	12.71, 0.99	0.03, 0.17	0.99
		Weighted median	1.11 (0.90 to 1.37)	0.33			
		MR−Egger	0.87 (0.63 to 1.19)	0.38			
CES	27	IVW	1.03 (0.91 to 1.17)	0.59	23.59, 0.60	0.005, 0.76	0.59
		Weighted median	1.05 (0.88 to 1.26)	0.56			
		MR−Egger	1.00 (0.78 to 1.28)	1.00			
SVS	26	IVW	1.15 (0.95 to 1.38)	0.15	41.02, 0.02	−0.03, 0.21	0.02
		Weighted median	1.14 (0.92 to 1.42)	0.22			
		MR−Egger	1.40 (0.98 to 2.01)	0.08			
All ICH	14	IVW	0.68 (0.40 to 1.15)	0.15	23.27, 0.054	0.08, 0.25	0.051
		Weighted median	0.54 (0.31 to 0.95)	0.03			
		MR−Egger	0.37 (0.13 to 1.11)	0.10			
Lobar ICH	13	IVW	0.72 (0.35 to 1.49)	0.37	27.19, 0.01	0.09, 0.39	0.71
		Weighted median	0.49 (0.23 to 1.03)	0.06			
		MR−Egger	0.39 (0.08 to 1.82)	0.26			
Deep ICH	13	IVW	0.59 (0.35 to 0.99)	0.048	9.04, 0.70	0.003, 0.97	0.40
		Weighted median	0.53 (0.26 to 1.08)	0.08			
		MR−Egger	0.57 (0.19 to 1.74)	0.35			
SAH	8	IVW	0.97 (0.69 to 1.37)	0.88	11.39, 0.12	0.06, 0.32	0.14
		Weighted median	0.91 (0.63 to 1.32)	0.61			
		MR−Egger	0.56 (0.20 to 1.59)	0.32			

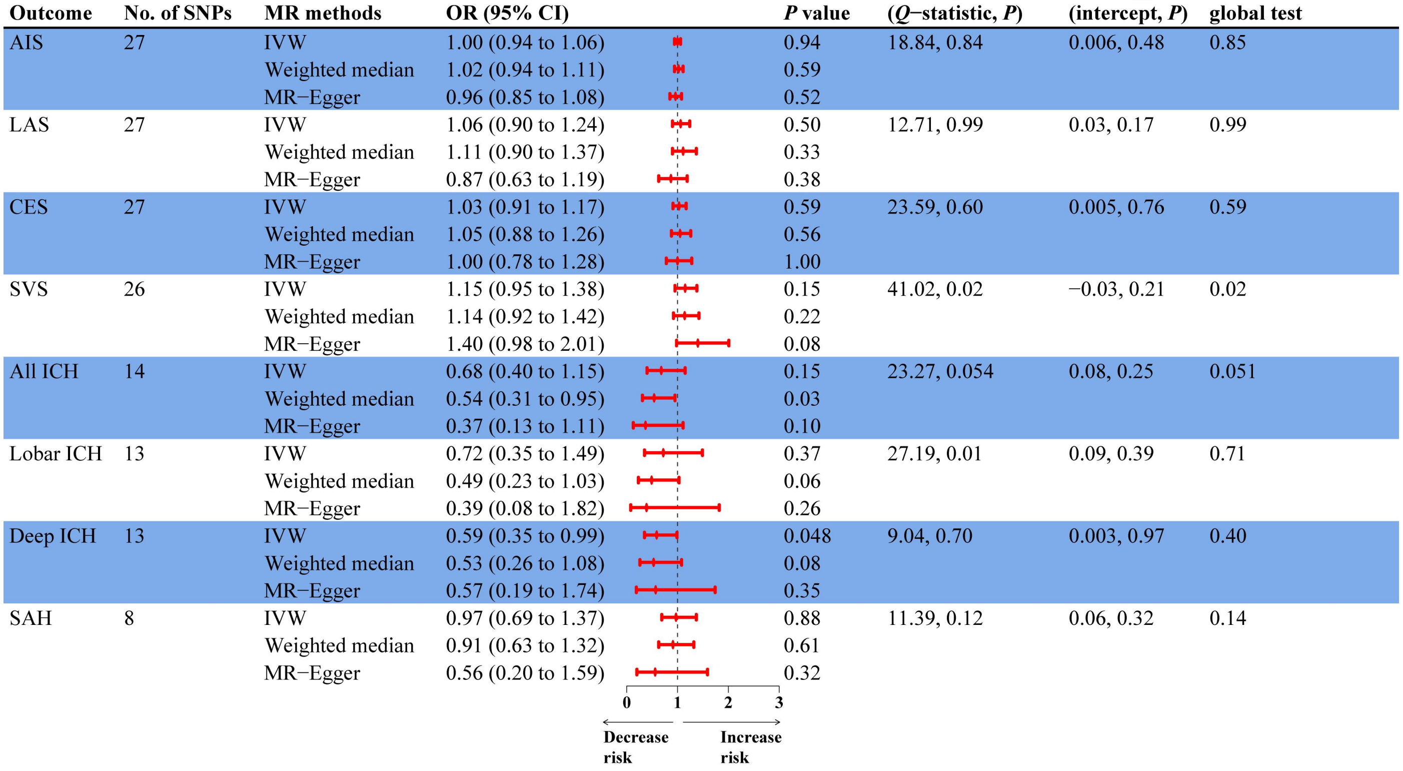

Figure S6. MR estimates from each method of assessing the causal effects of P4 on the risk of stroke subtypes. P4, progesterone; AIS, any ischemic stroke; LAS, large artery stroke; CES, cardioembolic stroke; SVS, small vessel stroke; ICH, intracerebral hemorrhage; SAH, subarachnoid hemorrhage; SNPs, single nucleotide polymorphisms; MR, Mendelian randomization; IVW, inverse variance weighted; OR, odd ratio; CI, confidence interval; MR-PRESSO, MR pleiotropy residual sum and outlier.

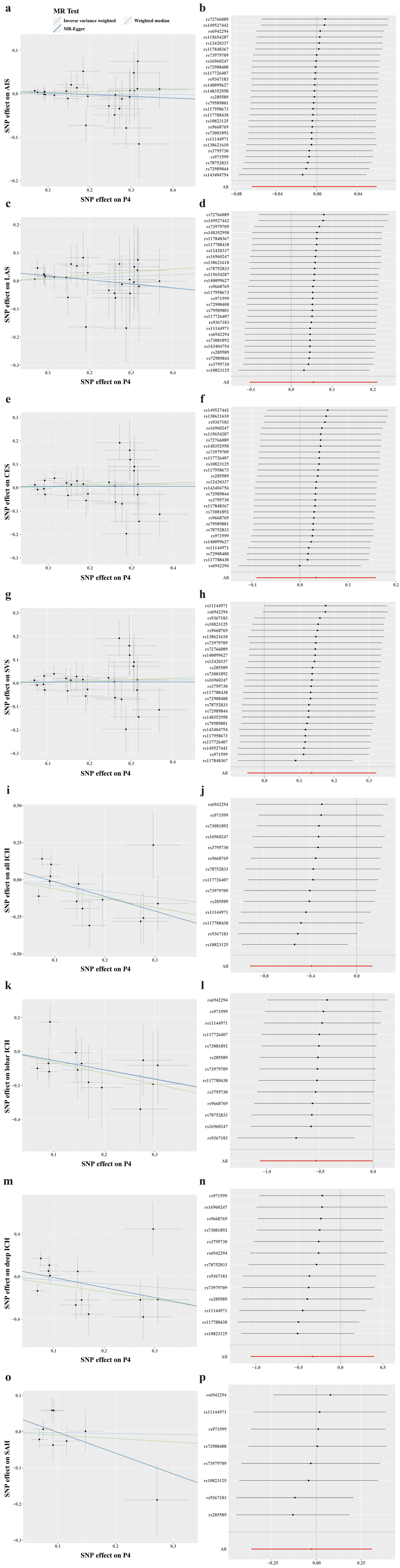

Figure S7. Scatter plot and leave-one-out test for genetically determined P4 and risk of stroke subtypes. (a, b) AIS, any ischemic stroke; (c, d) LAS, large artery stroke; (e, f) CES, cardioembolic stroke; (g, h) SVS, small vessel stroke; (i, j) all ICH, intracerebral hemorrhage; (k, l) lobar ICH; (m, n) deep ICH; (o, p) SAH, subarachnoid hemorrhage. P4, progesterone; SNP, single nucleotide polymorphism.

Outcome	No. of SNPs	MR methods	OR (95% CI)	*P* value	Heterogeneity test (*Q*−statistic, *P*)	MR−Egger (intercept, *P*)	*P* for MR−PRESSO global test
AIS	10	IVW	0.91 (0.82 to 1.01)	0.07	10.31, 0.33	0.009, 0.51	0.34
		Weighted median	0.96 (0.83 to 1.10)	0.54			
		MR−Egger	0.83 (0.63 to 1.09)	0.22			
LAS	10	IVW	0.79 (0.62 to 1.01)	0.06	4.89, 0.84	−0.01, 0.65	0.86
		Weighted median	0.80 (0.58 to 1.10)	0.17			
		MR−Egger	0.91 (0.49 to 1.66)	0.76			
CES	10	IVW	0.90 (0.74 to 1.09)	0.27	2.94, 0.97	−0.01, 0.66	0.97
		Weighted median	0.83 (0.66 to 1.05)	0.13			
		MR−Egger	1.00 (0.61 to 1.63)	0.99			
SVS	10	IVW	1.05 (0.81 to 1.35)	0.71	11.10, 0.27	0.00, 0.93	0.28
		Weighted median	1.05 (0.77 to 1.44)	0.76			
		MR−Egger	1.02 (0.52 to 2.00)	0.96			
All ICH	5	IVW	0.53 (0.23 to 1.23)	0.14	6.06, 0.19	0.58, 0.25	0.26
		Weighted median	0.57 (0.21 to 1.52)	0.26			
		MR−Egger	0.00 (0.00 to 12.59)	0.23			
Lobar ICH	5	IVW	0.63 (0.27 to 1.49)	0.30	4.42, 0.35	0.66, 0.23	0.35
		Weighted median	0.45 (0.14 to 1.39)	0.16			
		MR−Egger	0.00 (0.00 to 11.35)	0.22			
Deep ICH	6	IVW	0.48 (0.20 to 1.19)	0.11	5.99, 0.31	−0.12, 0.39	0.40
		Weighted median	0.66 (0.22 to 1.94)	0.45			
		MR−Egger	2.11 (0.09 to 50.27)	0.67			
SAH	5	IVW	1.05 (0.74 to 1.49)	0.80	3.33, 0.50	0.05, 0.37	0.53
		Weighted median	1.24 (0.78 to 1.97)	0.36			
		MR−Egger	0.53 (0.14 to 1.97)	0.41			

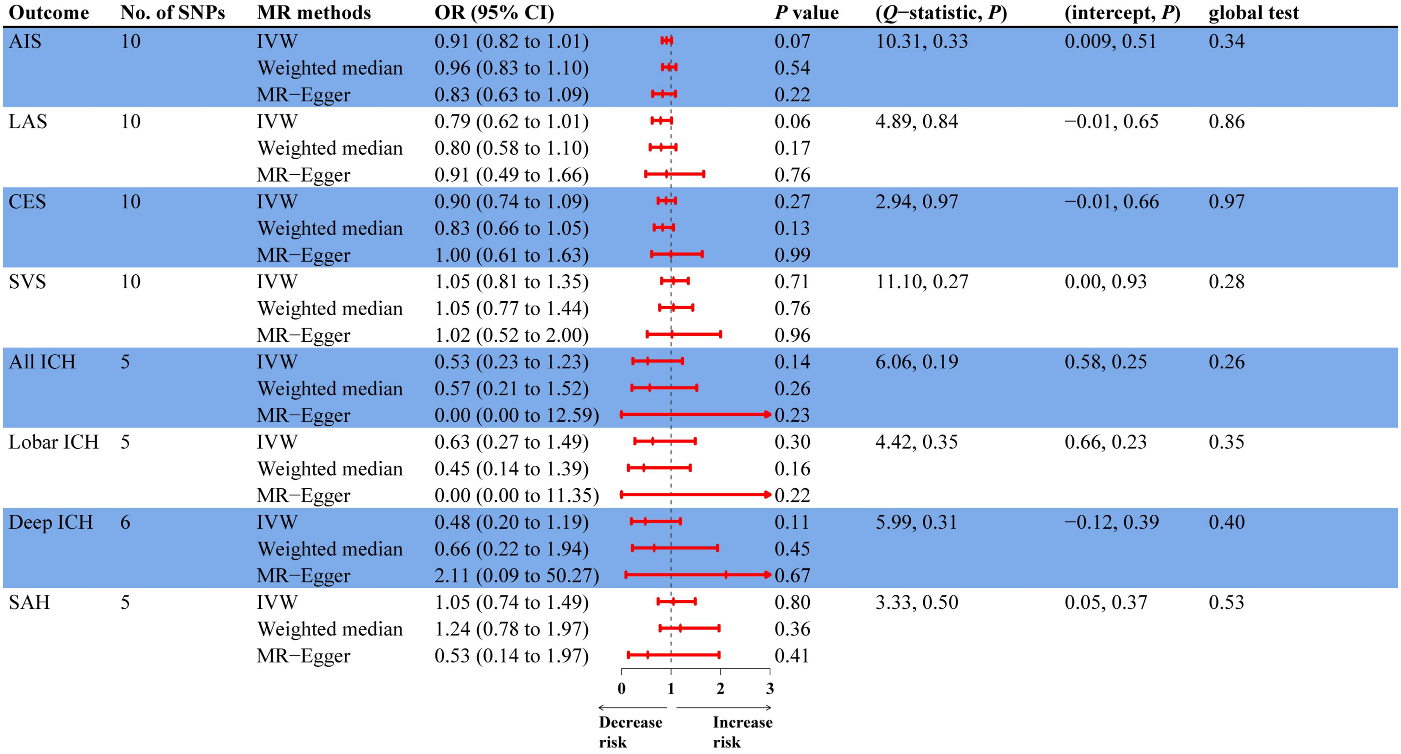

Figure S8. MR estimates from each method of assessing the causal effects of 17-OHP on the risk of stroke subtypes. 17-OHP, hydroxyprogesterone; AIS, any ischemic stroke; LAS, large artery stroke; CES, cardioembolic stroke; SVS, small vessel stroke; ICH, intracerebral hemorrhage; SAH, subarachnoid hemorrhage; SNPs, single nucleotide polymorphisms; MR, Mendelian randomization; IVW, inverse variance weighted; OR, odd ratio; CI, confidence interval; MR-PRESSO, MR pleiotropy residual sum and outlier.

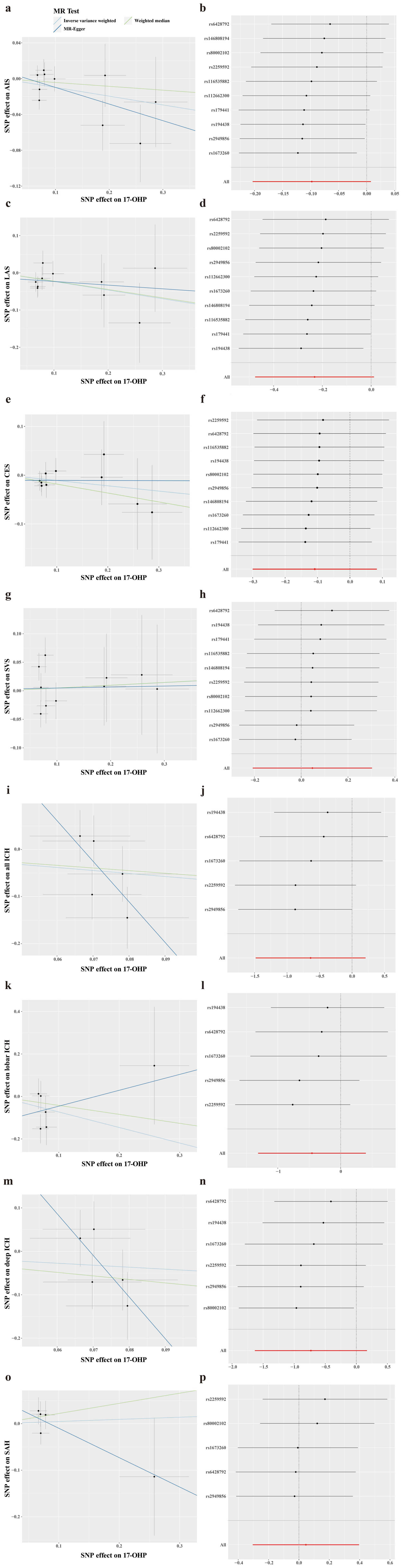

Figure S9. Scatter plot and leave-one-out test for genetically determined 17-OHP and risk of stroke subtypes. (a, b) AIS, any ischemic stroke; (c, d) LAS, large artery stroke; (e, f) CES, cardioembolic stroke; (g, h) SVS, small vessel stroke; (i, j) all ICH, intracerebral hemorrhage; (k, l) lobar ICH; (m, n) deep ICH; (o, p) SAH, subarachnoid hemorrhage. 17-OHP, hydroxyprogesterone; SNP, single nucleotide polymorphism.

Exposure	No. of SNPs	MR methods	OR (95% CI)	*P* value	Heterogeneity test (*Q*−statistic, *P*)	MR−Egger (intercept, *P*)	*P* for MR−PRESSO global test
T/E2 ratio	11	IVW	1.23 (1.02 to 1.47)	0.009	12.84, 0.23	0.03, 0.64	0.26
		Weighted median	1.19 (0.96 to 1.47)	0.12			
		MR−Egger	1.02 (0.65 to 1.61)	0.93			
Aldo	17	IVW	1.14 (0.93 to 1.39)	0.21	30.05, 0.12	0.03, 0.78	0.06
		Weighted median	1.11 (0.89 to 1.40)	0.35			
		MR−Egger	1.06 (0.61 to 1.84)	0.84			
A4	15	IVW	0.83 (0.67 to 1.01)	0.07	12.80, 0.54	0.02, 0.30	0.57
		Weighted median	0.80 (0.60 to 1.07)	0.13			
		MR−Egger	1.06 (0.64 to 1.75)	0.82			
P4	24	IVW	1.03 (0.89 to 1.20)	0.67	30.79, 0.13	0.02, 0.06	0.13
		Weighted median	0.95 (0.78 to 1.15)	0.62			
		MR−Egger	1.36 (1.03 to 1.79)	0.04			
17−OHP	9	IVW	0.99 (0.80 to 1.23)	0.95	8.21, 0.41	0.03, 0.79	0.40
		Weighted median	1.00 (0.75 to 1.33)	0.99			
		MR−Egger	1.08 (0.57 to 2.06)	0.82			

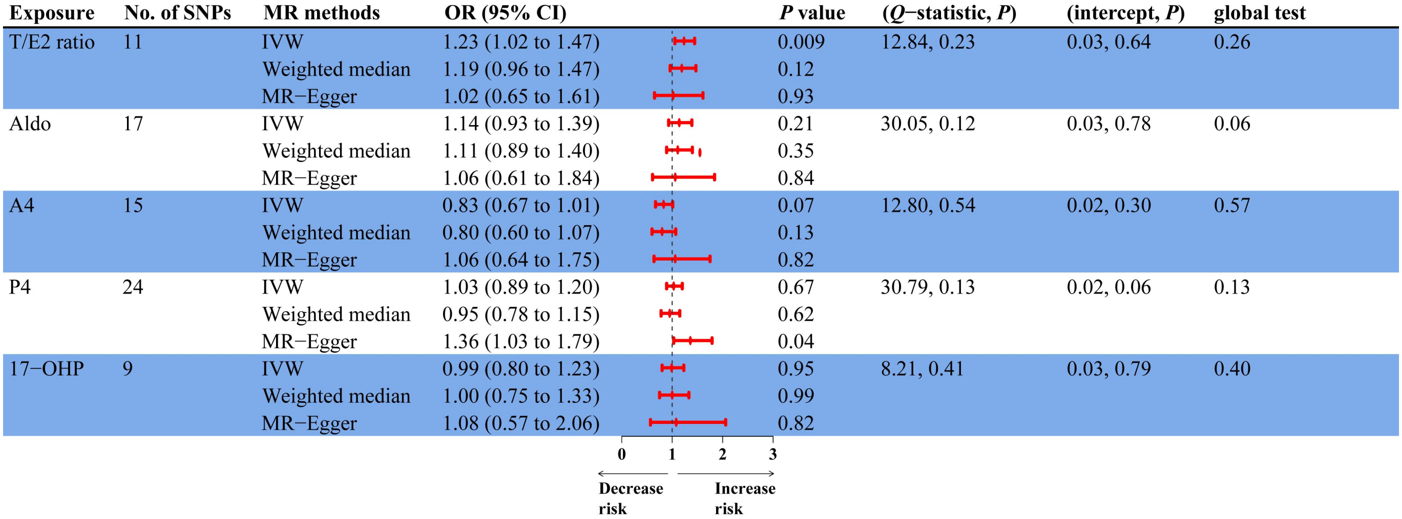

Figure S10. MR estimates from each method of assessing the causal effects of steriod on the risk of SVS in replicated stage. SVS, small vessel stroke; T/E2, testosterone/17β-estradiol; Aldo, aldosterone; A4, androstenedione; P4, progesterone; 17-OHP, hydroxyprogesterone; SNPs, single nucleotide polymorphisms; MR, Mendelian randomization; IVW, inverse variance weighted; OR, odd ratio; CI, confidence interval; MR-PRESSO, MR pleiotropy residual sum and outlier.

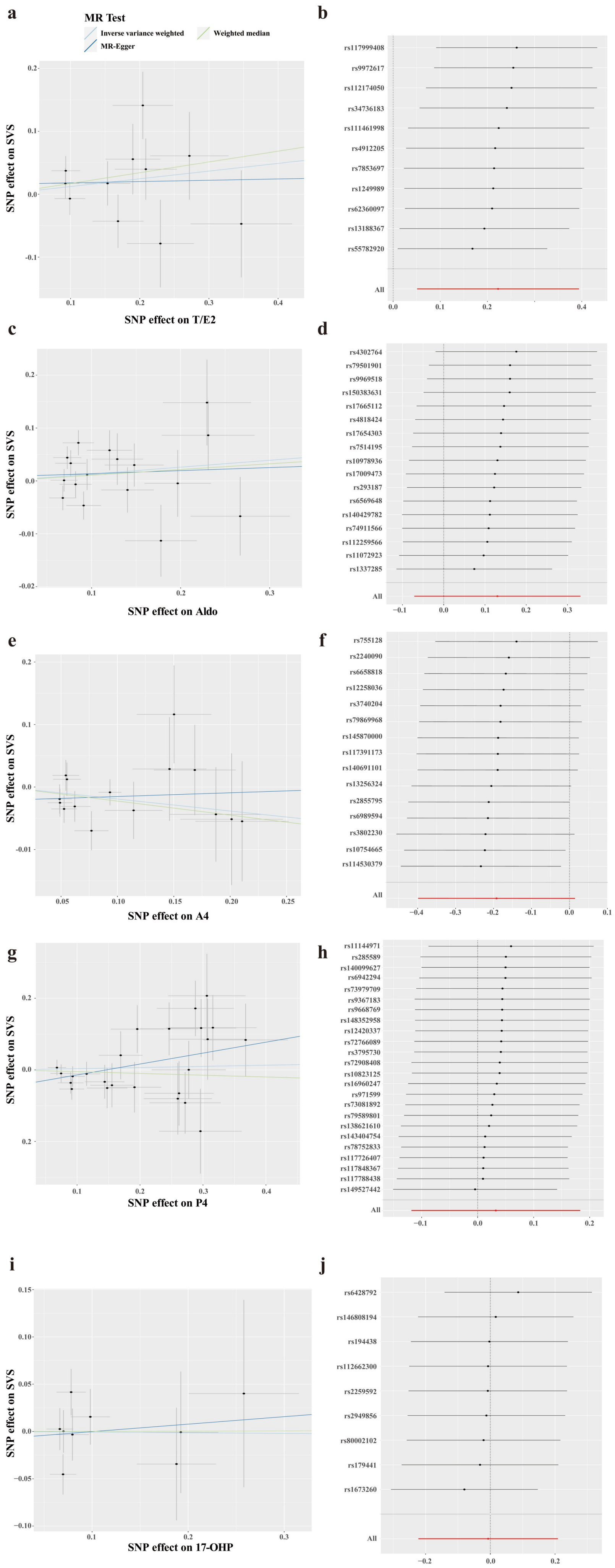

Figure S11. Scatter plot and leave-one-out test for genetically determined steroid harmonies and risk of SVS in replicated stage. (a, b) T/E2, testosterone/17β-estradiol ratio; (c, d) Aldo, aldosterone; (e, f) A4, androstenedione; (g, h) P4, progesterone; (i, j) 17-OHP, hydroxyprogesterone. SVS, small vessel stroke; SNP, single nucleotide polymorphism.
